# Myocardial Motion Analysis for Determination of Tei-Index of Human Heart

**DOI:** 10.3390/s101211428

**Published:** 2010-12-13

**Authors:** Shengyong Chen, Jianhua Zhang, Houxiang Zhang, Qiu Guan, Yahui Du, Chunyan Yao, Jianwei Zhang

**Affiliations:** 1 College of Computer Science, Zhejiang University of Technology, 310023 Hangzhou, China; 2 Department of Informatics, University of Hamburg, 22527 Hamburg, Germany

**Keywords:** cardiac cycle physical phase, Tei index, left ventricular wall motion amplitude, computer vision, image processing

## Abstract

The Tei index, an important indicator of heart function, lacks a direct method to compute because it is difficult to directly evaluate the isovolumic contraction time (ICT) and isovolumic relaxation time (IRT) from which the Tei index can be obtained. In this paper, based on the proposed method of accurately measuring the cardiac cycle physical phase, a direct method of calculating the Tei index is presented. The experiments based on real heart medical images show the effectiveness of this method. Moreover, a new method of calculating left ventricular wall motion amplitude is proposed and the experiments show its satisfactory performance.

## 1. Introduction

Cardiovascular diseases have become the top factor causing human death in both western and eastern world. People hope that these diseases can be traced before they onset. A simple, reproducible, non-invasive test for determinants of prognosis is therefore necessary. For this kind of test, doctors need to observe the current status of hearts and have some effective measurement rules to decide if hearts is normal or not. For non-invasive check methods, there are several heart medical imaging technologies, such as MR, CT, SPECT and Ultrasound, widely used for heart diseases diagnosis, from which doctors can observe patients heart status without invasion.

Myocardial motion that is directly related to cardiac vascular supply is widely studied based on heart medical images to analyze the heart condition, especially the left ventricular function, for diagnosing heart abnormalities [1–3]. Left ventricular (LV) is the most important part of heart and LV function is generally analyzed to diagnose the heart condition. Traditionally, LV systolic function is used as measurement of the heart condition. However, LV diastolic function is also an important indicator of the heart condition. Both of them are important determinants of prognosis. A clinical assessment of using both LV systolic and diastolic function will be better than using only one of them. The Tei index [4], raised originally by Tei in 1995, can be used to assess both LV systolic and diastolic function at the same time and consequently becomes an important indicator of heart condition [5,6]. In [7], several literatures had been reviewed in which the Tei index had been proved to be used to diagnose many cardiovascular diseases. The Tei index can be used to assess either left or right ventricular function, calculated as the sum of the isovolumic contraction time (ICT) and isovolumic relaxation time (IRT) divided by ejection time (ET). The ICT and IRT, however, are very difficult to calculate because of the lack of the whole deform information of LV. The Tei index is therefore generally measured by indirect methods (e.g., [8,9]. The originally method to evaluate the time intervals is through pulsed-wave Doppler velocity spectra of ventricular inflow and outflow [4]. However, there is possible error source because of the inability to acquire inflow and outflow velocity spectra during the same cardiac cycle [5]. The tissue Doppler imaging is employed to measure the time intervals for computing the Tei index to avoid this kind of potential error [9]. Another method using left ventricular area waveforms with acoustic quantification is also introduced to calculate the Tei index for avoiding the potential error. However, both of them are also the indirect methods.

In the myocardial motion analysis, the cardiac cycle is a complicated time-varying process and is generally partitioned into several phases based on time to simplify the process. According to clinical diagnosis, however, the phase should be definitely based on LV deformation and stress since the dynamics of heart is caused by that deformation. This definition is called cardiac physical phase. In this paper, a method of cardiac cycle physical phase (CPP) division according to the LV anatomy is proposed first based on B-spline represented heart model, of which the initial work [10] has been presented in Robio 2010. By using the proposed method, the CPP can be accurately partitioned. Based on accurately CPP partitions, a direct method to accurately measure the Tei index is then presented by computing the ICT and IRT from CPP. To our knowledge, it is the first direct calculation of the Tei index in literatures. In particular, the SPECT heart images are used in this study. As a kind of new application of heart medical images, the proposed calculation of the Tei index is proved better than traditional assessment methods.

Moreover, LV wall motion amplitude (LVMA) can also be computed to analyze the LV wall movement based on the accurate CPP partitions in this study. The movement of LV wall is another very important index of the LV function [11,12]. It reflects the deformation capability of LV. Traditionally, the analysis of LV wall movement is focused on myocardial velocities [13,14]. The LVMA, however, can indicate directly the abnormal area of LV wall when there is heart disease [15]. Through LVMA, the ill area of heart with abnormal amplitude could be detected and localized, which will of course improve the effect of diagnosis.

The organization of this paper is as follows. In the next section, the matrix representation of B-spline is introduced and the volumetric measurement of B-spline is given. The method of CPP partition and calculation of Tei and LVMA are explained in Section 3. The proposed methods are evaluated by experiments on a real human data in Section 4 and the results are also analyzed.

## 2. Shape Representation for Volumetric Measurement

### 2.1. Matrix representation of B-spline

A B-spline curve consists of segments, with each segment of degree k constructed by k+1 sequential control points. Let *t* = (*u*–*u_i_*)/(*u_i_*_+1_ –*u_i_*), the segment has the following matrix form [16,17] in the nonempty interval [*u_i_*,*u_i_*_+1_]:

(1) $$p_{i}(t)=\left[1\;\;\;\;\;t\;\;\;\;\;t^{2}\;\;\;\cdots \;\;\;t^{k}\right]M_{i}^{k+1}{\left[V_{i-k}\;\;\;\;\;V_{i-k+1}\;\;\;\cdots \;\;\;V_{i}\right]}^{T}$$

where *t* ∈ [0,1] and *V* is the vector of *k*+1 control points. The number of control points are denoted as *i* = *k*,*k*+1, ⋯,*n*+1 and the *i*th basis matrix, $M_{i}^{k+1}$, is the matrix form of B-spline basis in order *k*.

The whole curve can thus be represented as:

(2) $$\begin{array}{l} P(t)=\sum_{i=k}^{n}p_{i}(t)=\sum_{i=k}^{n}\sum_{j=0}^{k}a_{i}(j)t^{j}\\ \;\;\;\;\;\;\;\;\;\;=\sum_{i=k}^{n}\sum_{j=0}^{k}(\sum_{l=0}^{k}M_{i}^{k+1}(j,\;l)V_{i-k+l})t^{j} \end{array}$$

where

(3) $$a_{i}(j)=\sum_{l=0}^{k}M_{i}^{k+1}(j,\;l)V_{i-k+l}$$

Equally, a part of the B-spline surface in the nonempty area [*u_i_,u_i_*_+1_) × [*v_i_*,*v_i_*_+1_) can be represented as:

(4) $$s_{i,j}(t,\;w)=T_{k_{1}}M_{i,u}^{k_{1}+1}V_{i,j}^{h}{\left(M_{j,v}^{k_{2}+1}\right)}^{T}W_{k_{2}}^{T}$$

where *T_k_*__1__ = [1 *t t*^2^ ⋯ *t*^*k*_1_^] and $W_{k_{2}}^{T}={\left[1\;\;\;\;\;w\;\;\;\;\;w^{2}\;\;\;\cdots \;\;\;w^{k_{2}}\right]}^{T}$ with *t* = (*u*–*u_i_*)/(*u_i_*_+1_ –*u_i_*) and *w* = (*v*–*v_i_*)/(*v_i_*_+1_ –*v_i_*), where *u*,*v* ∈ [*u_i_*,*u_i_*_+1_) × [*v_i_*,*v_i_*_+1_). $M_{i,u}^{k_{1}+1}$ and $M_{j,v}^{k_{2}+1}$ are the *i*th and *j*th basis matrixes in [*u_i_*,*u_i_*_+1_) and [*v_i_*,*v_i_*_+1_), respectively.

The whole surface is then represented as follow:

(5) $$\begin{array}{l} X(t,\;w)=\sum_{i=k_{1}}^{m}\sum_{j=k_{2}}^{n}\sum_{l=0}^{k_{2}}\sum_{r=0}^{k_{1}}B_{i,j}^{x}(r,\;l)t_{r}w_{l}\\ Y(t,\;w)=\sum_{i=k_{1}}^{m}\sum_{j=k_{2}}^{n}\sum_{l=0}^{k_{2}}\sum_{r=0}^{k_{1}}B_{i,j}^{y}(r,\;l)t_{r}w_{l}\\ Z(t,\;w)=\sum_{i=k_{1}}^{m}\sum_{j=k_{2}}^{n}\sum_{l=0}^{k_{2}}\sum_{r=0}^{k_{1}}B_{i,j}^{z}(r,\;l)t_{r}w_{l} \end{array}$$

### 2.2. Volumetric measurement

The volume can be expressed as [18]:

(6) $$V=\int_{z_{1}}^{z_{2}}A(Z)dz$$

where *A*(*z*) is the area of the slice at depth *z*.

As the equations given by Equation 1 to Equation 4, let $\frac{\partial S(t,\;w)}{\partial t}$ and $\frac{\partial S(t,\;w)}{\partial w}$ denote the derivatives of *S*(*u*,*v*) with respect to *t* and *w*.

The computation of the area of a slice at depth *z* is:

(7) $$A(z(w))=\sum_{j=k_{2}}^{n}\int_{0}^{1}Y(t,\;w)\times \frac{\partial X(t,\;w)}{\partial t}dt$$

where *Y*(*t*,*w*) is given by Equation 6, and $\frac{\partial X(t,\;w)}{\partial t}$ is the derivative with respect to *t* and $\frac{\partial Z(t,\;w)}{\partial w}$ is the derivative with respect to *w*.

The volume of B-spline surface of orders *k*_1_ × *k*_2_ is:

(8) $$V=\sum_{i=k_{1}}^{m}\sum_{j=k_{2}}^{n}\left(\sum_{l_{1}=1}^{\left(3\times k_{2}-1\right)}\sum_{l_{2}=1}^{\left(3\times k_{1}-1\right)}C_{i,j}(l_{1},\;l_{2})\times \frac{1}{l_{2}+1}\times \frac{1}{l_{1}+1}\right)$$

where *C_i_*_,_ *_j_* is the matrix of size 3*k*_1_ × 3*k*_2_ which is the product of the three polynomials *Y*(*t*,*w*).

## 3. CPP Partition

### 3.1. CPP Partition

Cardiac cycle is a very complicated physical process. Traditional researches used to divide the cycle into two major parts as systole and diastole. During systole, LV contracts thus the blood in LV can be ejected out. On the opposite, during diastole LV bulges and imbibes blood in. The function of heart is achieved by the repeatedly alternating systole and diastole. As the LV motion is not uniform neither in systole nor in diastole, this rough partition can hardly express the true feature of LV motion. In fact it is shown by anatomy knowledge that the whole cardiac cycle contains 7 CPPs based on different LV shape and function. These CPPs are Isovolumic Systolic (IS), Rapid Ejection Period (REP), Slow Down Ejection Period (SDEP), Isovolumic Relaxation Period (IRP), Rapid Filling Period (RFP), Slow Down Filling Period (SDFP) and Atrial Systole (AS) [19] where IS, REP and SDEP compose the systole and IPR, RFP, SDFP belong to the diastole.

The shape of LV shows various features in different CPP, and the changing of these shapes is the main basis of CPP partition. To divide the CPP phases, two shape factors are necessary. The first shape factor is the volume of LV (LVV). Assume that the LVV curve during whole cardiac cycle is already known and denoted with *C*(*t*). It is easy to understand that during the whole systole *C*(*t*) keeps decreasing, while in diastole it keeps increasing. Let curve *C*′(*t*) denote derivative of *C*(*t*). According to medical knowledge, the features of *C*(*t*) and *C*′(*t*) in each CPP can be listed as in the columns 3–4 of Table 1. During the IS, IRP and AS phases, the values of *C*(*t*) remain almost invariant and consequently its derivative is approximate to zero. During the REP and SDEP phases, the values of *C*(*t*) keep on reducing and *C*′(*t*) is of course negative. During the RFP and SDFP phases, the values of *C*(*t*) keep on increasing and *C*′(*t*) is positive. The IS and AS, REP and SDEP, RFP and SDFP, however, are the adjacent phases, therefore it is difficult to separate them.

Fortunately, there is another shape factor, called LV long-axis length (LVLL), that can provide the complement information. LVLL is defined as the height from the base to the apical of LV. Assuming the LVLL curve is *L*(*t*), and its derivative is *L*′(*t*). According to the anatomy priori knowledge the *L*(*t*) and *L*′(*t*) features in each CPP are listed in the columns 5–6 of Table 1. During IS and SDFP phases, *L*(*t*) keeps on decreasing and *L*′(*t*) is negative. During SDEP phase, *L*(*t*) keeps on increasing and *L*′(*t*) is positive. By compounding the shape factors, LVV and LVLL, it is now easy to divide the seven phases of cardiac cycle. Figure 2 illustrate examples of *C*′(*t*) and *L*′(*t*).

### 3.2. Determination of Tei index

In the beginning of systole and diastole, the shape of LV sharply changes while the LVV stays invariant. These periods are called isovolumetric contraction (IC) and isovolumetric relaxation (IR). Medical research shows that the biggest stress and displacement of LV occur in these two periods. When some diseases occur, the LV would need longer time to achieve the deformation and displacement, thus the length of IC and IR are very important index for evaluating LV functions. Based on that Tei-index is raised by Japanese researcher Tei in 1995 [4]. The definition of Tei is:

(9) $$\text{Tei}\;\text{index}=\frac{t_{IC}+t_{IR}}{t_{E}}$$

Tei index is also called myocardial performance index (MPI). This new index can generally evaluate the heart function in both diastole and systole. It has been widely used in heart disease diagnosis. Based on the CPP partition, a direct method of calculating Tei index can be deduced.

IC and IR are the periods while the LV shape changes largely and LVV keeps almost invariant. According to the CPP characters listed in Table 1, it is obvious that IC is equal to IS and IR is equal to IRP. The ET is the length of systole which equals to the sum of IS, REP and SDEP.

The Tei index can therefore be calculated as:

(10) $$\text{Tei}\;\text{index}=\frac{t_{IS}+t_{IRT}}{t_{IS}+t_{PRE}+t_{SDEP}}$$

where the *t_x_* is the length of time intervals of the corresponding CPP with *x* (*x* = IS, IRT, PRE, and SDEP). This equation is totally based on the partitions of CPP. Due to the accurate CPP partitions, the precise and direct calculation of Tei index can obtained.

### 3.3. Myocardial Motion

The function of LV is achieved by cycle motion of LV wall. The motion amplitude of LV wall (LVMA) reflect the deformation capability of LV. When some disease occurs, there must be some abnormalities of the LVMA on the sick area, thus LVMA can be used as an important parameter for LV function analysis. Azhari *et al.* [15] have used LVMA in the detection of dysfunctional myocardium.

It is also known that the movement of LV wall is not in a uniform speed, thus the distribution of LVMA differs largely in different CPPs. For example, the LVMA in the beginning of systole and diastole are much larger than it in other time because the main deformation of LV happens in these periods. Due to these causes mentioned above, to study on the LVMA in different CPP is highly necessary.

Assuming that the point *p* is on the wall of LV. By tracking the position of *p* at each time interval *t*, if the time of the whole cardiac cycle is *mt*, a position matrix can be obtained *T* = [*p*(*t*) *p*(2*t*) ⋯ *p*(*mt*)](*p*(*t*) = [*x_t_* *y_t_* *z_t_*]*^T^*). Fitting each row in T with three B-spline curves *M_x_*(*t*), *M_y_*(*t*) and *M_z_*(*t*) which stand for the trajectory of *p* in *x*, *y* and *z* axis during the whole cardiac cycle will be obtained. Through the trajectory curve of *p*, it is very simple to calculate the displacement of *p* in an arbitrary period [*t*_1_, *t*_2_] (0 ≤ *t*_1_ < *t*_2_ ≤ *mt*) by:

(11) $$D_{1,2}=\sqrt{{\left(M_{x}(t_{2})-M_{x}(t_{1})\right)}^{2}+{M_{y}(t_{2})-M_{y}(t_{1}))}^{2}+{M_{z}(t_{2})-M_{z}(t_{1}))}^{2}}$$

In order to get the displacement of whole LV wall, a large amount of points should be marked and tracked. Assuming such a points set *P* = [*p*_1_, *p*_2_, ⋯, *p_n_*] and their position matrix *T* are known, through Equation 11 the *D*_1,2_ of each point in *P* can be calculated. The displacement vector is $D=\left[D_{1,2}^{1},\;D_{1,2}^{2},\;\cdots \;,\;D_{1,2}^{n}\right]$, where $D_{1,2}^{i}$ means the displacement of *i*th point in *P*.

As an elastomer, the motion of LV is continuous and gradually variational so that the displacement of the whole LV wall can be described as a surface. Using the position information of *P*(*t*_1_) (position of all points in *P* at *t*_1_) and *D* a B-spline surface can be fitted. This surface is the displacement of whole LV wall in period [*t*_1_, *t*_2_] and be denoted by *Df* (*t*_1_, *t*_2_).

In Section 1 the cardiac cycle is divided into 7 CPPs (*i.e.*, IS, REP, SDEP, IRP, RFP, SDFP, AS). The beginning and ending time of each CPP is *tb_name_* and *te_name_*. Then the LVMA of each CPP can be calculated by *Df* (*tb_name_*, *te_name_*).

## 4. Experiments

### 4.1. LV B-spline Model

The 3D heart model is built by the Active Shape Models (ASM) [20] which has been extended to 3D representation in our previous work. By manually labeled landmarks which form the point distribution model in training images according to the rules described in [20], the ASM can learn the variations of heart shapes. The model can then be used to segment and fit a new heart in a set of new images. Note that the model is represented by a point set. After a new 3D heart is fitted by the model, those points of this heart model are used as the control points from which the B-spline heart model can be built according to Equation 1 to Equation 5.

**Figure 1.**
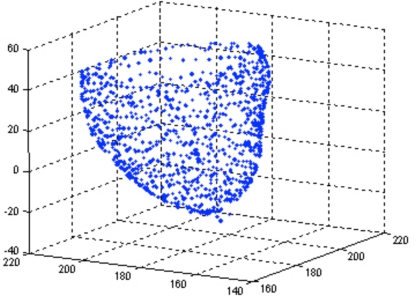
An example of the LV endocardium point model at one time phase.

Our experiment is focused on the left ventricle, about 1000 points have been tracked, and about 400 points are distributing on inside-wall while rests are on outside-wall. These points constitute the whole motion of left ventricle in a cardiac cycle, and the points are labeled in several phases, with 0.1 second apart from each other. In the model we focus on the inside-wall surface since the LVV and LVLL is majorly decided by it. Figure 1 shows one of the point model.

### 4.2. LVV and LVL curve interpolation

**Figure 2.**
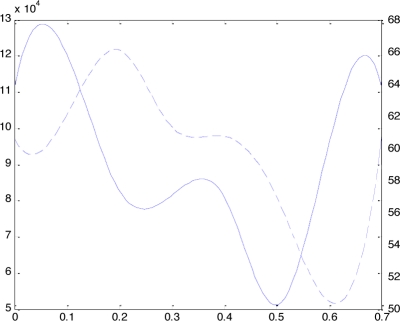
LVV varying cure (solid line) and LVLL varying cure (dash line) in whole cardiac cycle.

After the models in all phases are built, the LVV in each moments can be calculated by Equation 1. These LVV values are *v_i_* where *i* = 1,2, ⋯,7. Using the set of *v_i_* to interpolate with B-spline method then a LVV curve *C*(*t*) is fitted. The derivative *C*′(*t*) can also be calculated. The curve with solid line in Figure 2 is an example of the *C*′(*t*) curve. The landmarks of right Y-axis is corresponding to this curve.

In each inside-wall surface model, finding the centers at top and bottom layer, the LVLL can be calculated by the distance of these two centers. Assuming that the LVLL values are obtained from *m* model, *l_i_* where *i* = 1,2, ⋯, *m*, a B-spline curve *L*(*t*) can be fitted with *l_i_* and the derivative curve *L*′(*t*) can also be calculated. The curve with dash line in Figure 2 is an example of the *L*′(*t*) curve. The landmarks of left Y-axis is corresponding to this curve.

### 4.3. Measurement of CPP and Tei-index

With the curves of *C*′(*t*) and *L*′(*t*), the partition of CPP can be done by following steps.

**Figure 3.**
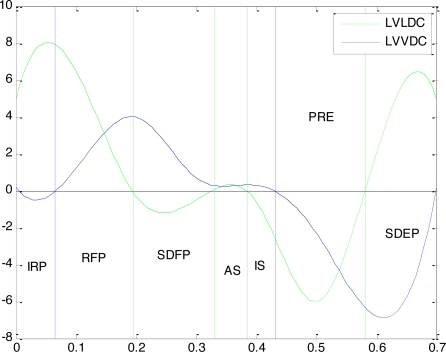
CPP partition in cardiac cycle.

Step 1: find the zero-points in *C*′(*t*) as the boundary of systolic and diastolic (blue line in Figure 3);
Step 2: find the zero-points in *L*′(*t*), to do the further division of CPP(green line in Figure 3).

The final CPP partition result is shown in Figure 3, and the lasting time of each CPP is calculated too. The comparison of the result to the medical statistical average data decided by experts is shown in Table 2 . Obviously the experiment result is very close to the average data which can prove the validity of this partition.

The Tei index can be calculated by Equation 10. In this experiment the value of Tei is 0.3617 while the common range of Tei is 0.39±0.05 by medical statistics.

**Figure 4.**
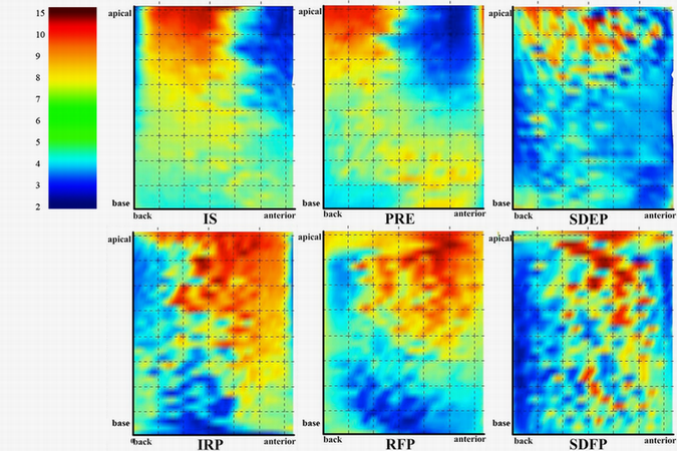
LVMA color graph of 6 CPPs. The color band in left column indicates the value of LVMA.

The distribution of LVMA in 6 CPPs is shown in Figure 4 (AS is neglected because in that phase the deformation of LV is inconspicuous). It can be seen that the LVMA of LV apical is much larger than that of LV base. During the systole LVMA concentrates in the back of LV while do the opposite during diastolic period. The largest LVMA occurs in IS and IRP which are the beginning of systole and diastole respectively. The result agrees with the medical statistical rule and is very closed to the result of other research [21,22].

## 5. Conclusions

In this paper, a new perspective of myocardial motion analysis, accurate division of cardiac cycle physical phases (CPP), is proposed. Based on this method, an precise and direct calculation of the Tei index is presented. First, several B-spline models of LV inside-wall are built at different time phases. Second, the LVV and LVLL of each model are calculated by B-spline integral and the curves of these two factors are fitted in whole cardiac cycle. Third, the derivative curves of LVV and LVLL are calculated. At last, the CPP partition by finding zero points in them is obtained. The experiment with real human LV data shows the result of this method is very close to the medical statistical values. Tei index is a new heart function index which strongly depends on the CPP. With the accurate CPP division, the accuracy of the direct Tei index calculation method is shown by the experiment result. This paper also proposed a new method to computing LVMA to analyze the myocardial motion. The color figure of LVMA in different CPP shows rules of myocardial motion which are very close to the results of other researches.

## Figures and Tables

**Table 1.** LVV and LVLL Features of Each CPP.

	*CPP*	*LVV* (*C*(*t*))	*C′*(*t*)	*LV LL*(*L*(*t*))	*L′*(*t*)
Systolic	IS	Hold on	*≈* 0	Reduce	*<* 0
	REP	Reduce	*<* 0	Reduce	*<* 0
	SDEP	Reduce	*<* 0	Increase	*>* 0
Diastolic	IRP	Hold on	*≈* 0	Increase	*>* 0
	RFP	Increase	*>* 0	Increase	*>* 0
	SDFP	Increase	*>* 0	Reduce	*<* 0
	AS	Hold on	*≈* 0	Hold on	*≈* 0

**Table 2.** CPP Lasting Time.

Phase	Average data	Result
IS	0.06–0.08	0.066
PRE	0.11	0.128
SDEP	0.14	0.132
IRP	0.06–0.08	0.057
RFP	0.11	0.047
SDFP	0.12	0.151
AS	0.1	0.119
